# Changes in compact bone microstructure of rats subchronically exposed to cadmium

**DOI:** 10.1186/s13028-014-0064-0

**Published:** 2014-09-24

**Authors:** Hana Duranova, Monika Martiniakova, Radoslav Omelka, Birgit Grosskopf, Ivana Bobonova, Robert Toman

**Affiliations:** Department of Zoology and Anthropology, Constantine the Philosopher University, 949 74 Nitra, Slovakia; Department of Botany and Genetics, Constantine the Philosopher University, 949 74 Nitra, Slovakia; Institute of Zoology and Anthropology, Georg-August University, 37 073 Göttingen, Germany; Department of Veterinary Sciences, Slovak University of Agriculture, 949 76 Nitra, Slovakia

**Keywords:** Compact bone, Cadmium, Rat, Osteoporosis

## Abstract

**Background:**

Chronic exposure to cadmium (Cd), even at low concentrations, has an adverse impact on the skeletal system. Histologically, primary and secondary osteons as basic structural elements of compact bone can also be affected by several toxicants leading to changes in bone vascularization and mechanical properties of the bone. The current study was designed to investigate the effect of subchronic peroral exposure to Cd on femoral bone structure including histomorphometry of the osteons in adult male rats.

In our study, 20 one-month-old male Wistar rats were randomly divided into two experimental groups. In the first group, young males received a drinking water containing 30 mg of CdCl_2_/L, for 90 days. Ten one-month-old males without Cd intoxication served as a control group. After 90 days of daily peroral exposure, body weight, femoral weight, femoral length, cortical bone thickness and histological structure of the femora were analysed.

**Results:**

We found that subchronic peroral application of Cd had no significant effect on body weight, femoral length and cortical bone thickness in adult rats. On the other hand, femoral weight was significantly increased (*P* < 0.05) in Cd-intoxicated rats. These rats also displayed different microstructure in the middle part of the compact bone where vascular canals expanded into central area of *substantia compacta* and supplied primary and secondary osteons. Additionally, a few resorption lacunae which are connected with an early stage of osteoporosis were identified in these individuals. Histomorphometrical evaluations showed that all variables (area, perimeter, maximum and minimum diameter) of the primary osteons’ vascular canals, Haversian canals and secondary osteons were significantly decreased (*P* < 0.05) in the Cd group rats. This fact points to alterations in bone vascularization.

**Conclusions:**

Subchronic peroral exposure to Cd significantly influences femoral weight and histological structure of compact bone in adult male rats. It induces an early stage of osteoporosis and causes reduced bone vascularization. Histomorphometrical changes of primary and secondary osteons allow for the conclusion that the bone mechanical properties could be weakened in the Cd group rats. The current study significantly expands the knowledge on damaging action of Cd on the bone.

## Background

Cadmium (Cd) is considered as one of the most toxic heavy metals [1]. Adverse effects of this non-essential metal are dependent on dose, route of administration, gestational age, species, and animal strain [2].

Bone is one of the target organs for Cd toxicity [3,4]. Various changes in the skeletal system characterized by osteopenia, osteoporosis, and/or osteomalacia with increased incidence of bone fractures belong to the main unfavorable health effects of chronic environmental and occupational exposure to Cd [5-7]. The results obtained by Brzóska and Moniuszko-Jakoniuk [8] have shown that chronic, even low-level exposure to Cd disturbs bone metabolism during skeletal development and maturity by affecting bone turnover most probably through a direct influence on bone formation and resorption, and indirectly via disorders in calcium (Ca) metabolism. Besides interfering with Ca, Cd also alters the metabolism of other metals essential for bone health [9]. For instance, Cd decreases zinc (Zn) concentration in bones and inhibits activities of Zn-dependent enzymes, such as alkaline phosphatase (it plays an important role in bone mineralization) [10,11] and superoxide dismutase (SOD, is significant in bone antioxidant defense system) [10]. Galicka *et al*. [12] have reported that receiving of 50 mg Cd/L in drinking water for six months influenced the collagen content and its solubility in the femoral bone of female rats. Any disturbances in collagen metabolism result in the formation of low-quality bone tissue susceptible to deformation and fractures [13].

The damaging action of Cd on the rat skeleton during chronic exposure and its possible mechanisms have been extensively studied and reported by other researches; however, a detailed histological analysis of compact bone including histomorphometrical evaluations of primary and secondary osteons after subchronic peroral administration of Cd in rats had not been done prior to our experiment. In general, individual osteon morphology reflects changes in formation and resorption of bone, thus histomorphometrical analyses may give important information about the reorganization process in the bone also in stress situation, e.g. intoxication with various xenobiotics. Therefore, the aim of the present study was to analyse changes in macroscopical structure of femoral bones, and also in qualitative and quantitative histological characteristics of compact bone in rats subchronically exposed to Cd in their drinking water. In the case of potential alterations, the hypothesis of toxic effects of Cd on femoral bone structure, mechanical properties and vascularization of investigated bones will be described.

## Methods

### Animals

Our experiment was conducted on 20 one-month-old male Wistar rats obtained from the accredited experimental laboratory (number SK PC 50004) of the Slovak University of Agriculture in Nitra. Generally, the laboratory rat is the preferred animal for most researchers. Its skeleton has been studied extensively in various experimental protocols leading to bone loss, including hormonal interventions, immobilization, and dietary manipulations. Although there are several limitations to its similarity to the human condition, these can be overcome through detailed knowledge of its specific traits or with certain techniques [14]. We used the males because they are less susceptible to skeletal damage than females [15-18]. The rats were housed individually in plastic cages under constant temperature (20–22°C), humidity (55 ± 10%), and 12/12 h cycle of light and darkness with access to food (feed mixture M3, Machal, Bonargo, Czech Republic) and drinking water *ad libitum*. We used cages Tecniplast 2154 F (Italy) which comply with the Government Regulation No. 23/2009. The cage disposed dimensions 480 × 265 × 210 mm, with a floor area 940 cm^2^. We used wood chips as bedding material with paper rolls which enriched the living-space of animals. The clinically healthy experimental animals were randomly divided into two groups, of ten animals each. In the first group (Cd group), young males were dosed with a daily Cd intake of 30 mg CdCl_2_/L in drinking water for 90 days. The second group (control group; n = 10) without Cd intoxication served as a control. The water consumption was daily monitored during the whole experiment. Since Cd absorption from the gastrointestinal tract in rats is lower than in humans [3], these animals need higher Cd doses to reach its concentration in the body similar to those noted in humans [19]. The concentration of Cd (30 mg CdCl_2_/L in drinking water; chosen on the basis of studied literature and our previous experiments with tested dose–response effects) was high enough to reach a toxicity level but also safe enough to prevent animal mortality (non-lethal dose) [3,4,18,20-22]. The daily dose of Cd in the Cd group was 0.596–0.597 mg/kg body weight (bw), which reflects the exposure in humans inhabiting moderately contaminated areas or via cigarette smoking (0.238–0.977 mg/kg bw) [18].

All procedures were approved by the Animal Experimental Committee of the Slovak Republic.

### Procedures

At the end of 90 days, all rats were euthanized, weighed and their femurs were used for macroscopical and microscopical analyses. The right femurs were weighed on analytical scales with an accuracy of 0.01 g and the femoral length was measured with a sliding instrument. For histological analyses, the right femurs were sectioned at the midshaft of the diaphysis and the segments were fixed in HistoChoice fixative (Amresco, USA). The segments were then dehydrated in increasing grades (40 to 100%) of ethanol and embedded in Biodur epoxy resin (Günter von Hagens, Heidelberg, Germany) according to the method described by Martiniaková *et al*. [23]. Transverse thin sections (70–80 μm) were prepared with a sawing microtome (Leitz 1600, Leica, Wetzlar, Germany) and fixed onto glass slides by Eukitt (Merck, Darmstadt, Germany) [24]. The qualitative histological characteristics of the compact bone were determined according to the internationally accepted classification systems of Enlow and Brown [25] and Ricqlés *et al*. [26], who classified bone tissue into three main categories: primary vascular tissue, non-vascular tissue and Haversian bone tissue. Various patterns of vascularization can occur in primary vascular bone tissue: longitudinal, radial, reticular, plexiform, laminar, lepidosteoid, acellular, fibriform and protohaversian. There are three subcategories identified in Haversian bone tissue: irregular, endosteal and dense. The quantitative (histomorphometrical) variables were assessed using the software Motic Images Plus 2.0 ML (Motic China Group Co., Ltd.). We measured area, perimeter and the minimum and maximum diameters of primary osteons’ vascular canals, Haversian canals and secondary osteons in all views (i.e., anterior, posterior, medial and lateral) of the thin sections in order to minimize inter-animal differences. Secondary osteons were distinguished from primary osteons (i.e., primary vascular canals) on the basis of the well-defined peripheral boundary (cement line) between the osteon and the surrounding tissue. Diaphyseal cortical bone thickness was also measured by Motic Images Plus 2.0 ML software. Twenty random areas were selected, and average thickness was calculated for each femur.

### Statistics

Statistical analysis was performed using SPSS 8.0 software. All data were expressed as mean ± standard deviation (SD). The unpaired Student’s *T*-test was used for establishing statistical significance (*P* < 0.05) between Cd and control groups of rats.

## Results

### Macroscopical differences

Body weight, femoral length and cortical bone thickness were unchanged in rats exposed to Cd as compared to the control group. On the contrary, femoral weight was significantly increased (*P* < 0.05) in Cd-intoxicated rats (Table 1).

**Table 1:** **Body weight, femoral weight, femoral length and cortical bone thickness in adult male rats subchronic exposed to 30 mg of CdCl**_**2**_ **/L in drinking water for 90 days (Cd group) and the control rats (control group)**

**Group**	**N**	**Body weight (g)**	**Femoral weight (g)**	**Femoral length (cm)**	**Cortical bone thickness (mm)**
Control group	10	405 ± 52.65	1.05 ± 0.17	3.94 ± 0.09	0.575 ± 0.048
Cd group	10	422.5 ± 27.21	1.27 ± 0.14	3.99 ± 0.14	0.573 ± 0.048
*T*-test		NS	t = 3.123	NS	NS
			df = 18		
			*P* = 0.006		

N: number of rats, NS: non-significant changes.

### Microscopical differences

**Figure 1:**
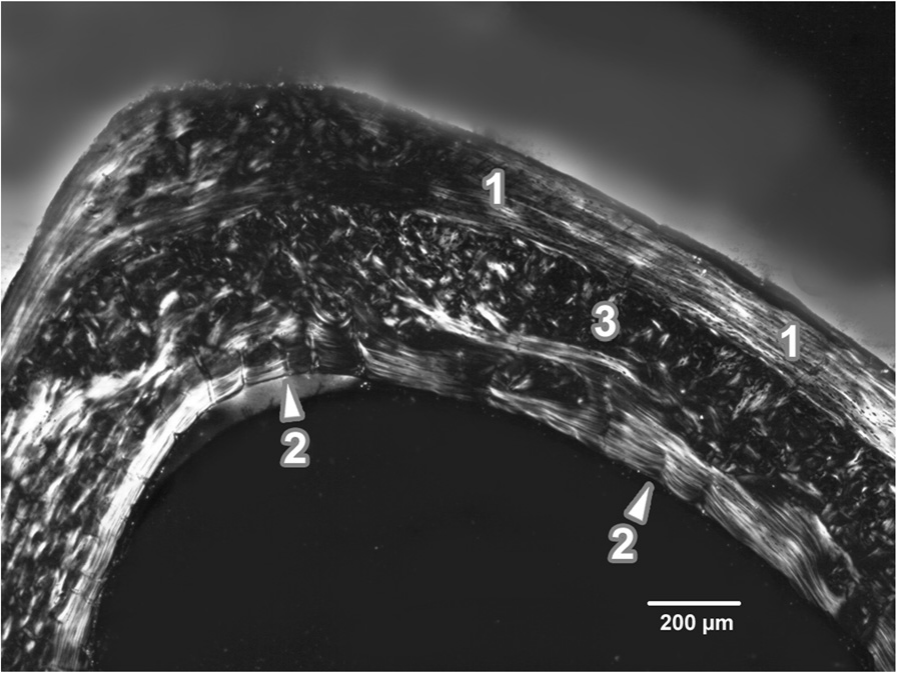
**Microscopic structure of compact bone in rat from the control group.** 1- non-vascular bone tissue. 2- vascular canals radiating from marrow cavity. 3- primary and secondary osteons in the middle part of compact bone.

Diaphysis of all femurs from the control rats had the following microstructure in common. Endosteal borders were formed by non-vascular bone tissue in all views (anterior, posterior, medial and lateral) of the thin sections. This type of bone tissue contained cellular lamellae and osteocytes without occurrence of primary and/or secondary osteons. Areas of primary vascular radial bone tissue (formed by branching or non-branching vascular canals radiating from the marrow cavity) were also identified in anterior, posterior and lateral views. Some primary and secondary osteons were exceptionally found in anterior and posterior views near endosteal surfaces. The occurrence of the osteons (primary and secondary) was also identified in middle parts of *substantia compacta*. The periosteal border was again composed of non-vascular bone tissue, mainly in the anterior and posterior views (Figure 1).

**Figure 2:**
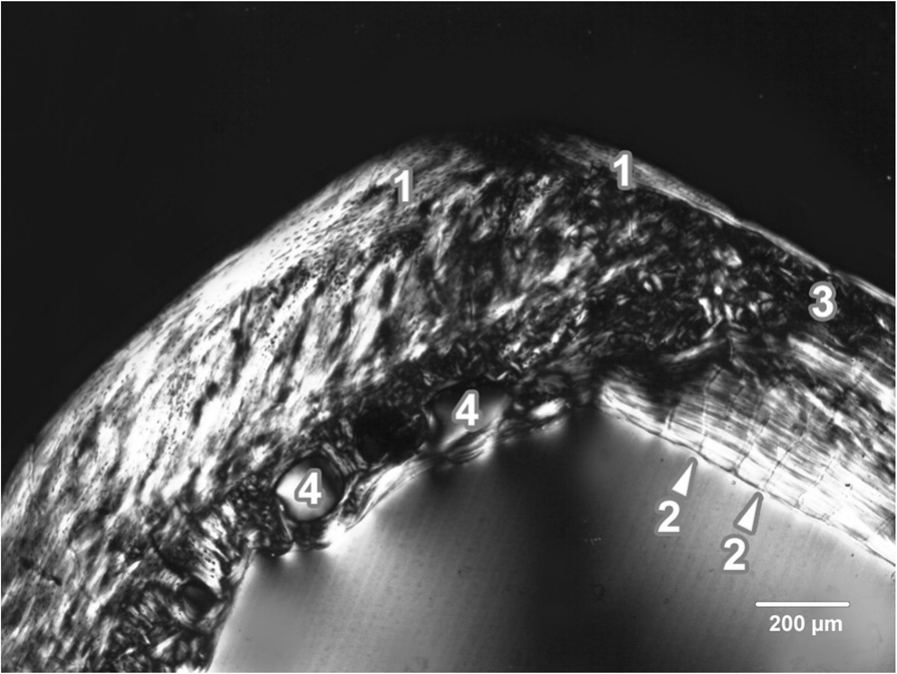
**Microscopic structure of compact bone in rat from the Cd group.** 1- non-vascular bone tissue. 2- enormous vascular canals radiating from marrow cavity. 3- smaller number of primary and secondary osteons in the middle part of compact bone. 4- resorption lacunae near endosteal surface.

The rats exposed to Cd displayed a similar microarchitecture to that of the control rats, except for the middle part of the compact bone in the medial and lateral views. In these views, primary vascular radial bone tissue occurred because vascular canals expanded from endosteal border into central area of the bone and supplanted primary and secondary osteons. Therefore, a smaller number of the osteons was observed in Cd-intoxicated rats. In these rats, we identified a few resorption lacunae near endosteal surfaces (mainly in antero-medial and postero-medial views) which are connected with an early stage of osteoporosis (Figure 2).

For the quantitative histological characteristics, 841 vascular canals of primary osteons, 435 Haversian canals and 435 secondary osteons were measured in total. The results are summarized in Table 2. We have found that subchronic peroral exposure to Cd significantly affects the sizes of primary and secondary osteons in male rats. All measured variables (area, perimeter, maximal and minimal diameter) of the primary osteons’ vascular canals, Haversian canals and secondary osteons were significantly lower (*P* < 0.05) in rats from the Cd group as compared to those of the control one.

**Table 2:** **Data of the primary osteons’ vascular canals, Haversian canals and secondary osteons in adult male rats subchronic exposed to 30 mg of CdCl**_**2**_ **/L in drinking water for 90 days (Cd group) and the control rats (control group)**

**Measured structure**	**Group**	**N**	**Area (μm** ^**2**^ **)**	**Perimeter (μm)**	**Max. diameter (μm)**	**Min. diameter (μm)**
**Primary osteons’ vascular canals**	Control group	436	393.49 ± 123.05	71.58 ± 12.37	12.87 ± 2.68	9.62 ± 1.68
	Cd group	405	341.39 ± 65.77	66.74 ± 7.27	11.77 ± 1.08	9.29 ± 1.13
	*T*-test		t = 7.732	t = 6.975	t = 7.007	t = 3.385
			df = 674.963	df = 712.451	df = 767.944	df = 768.604
			*P* = 0.001	*P* = 0.001	*P* = 0.001	*P* = 0.001
**Haversian canals**	Control group	208	428.3 ± 75.75	74.94 ± 7.3	13.04 ± 1.56	10.73 ± 1.17
	Cd group	227	384.94 ± 73.23	70.9 ± 6.94	12.52 ± 1.67	9.84 ± 1.31
	*T*-test		t = 6.403	t = 6.403	t = 3.533	t = 7.880
			df = 483	df = 483	df = 483	df = 483
			*P* = 0.001	*P* = 0.001	*P* = 0.001	*P* = 0.001
**Secondary osteons**	Control group	208	6408.74 ± 1820.97	292.98 ± 39.59	51.64 ± 8.17	39.51 ± 6.2
	Cd group	227	4583. 64 ± 1326.92	240.60 ± 35.32	41.50 ± 6.92	34.6 ± 5.32
	*T*-test		t = 12.467	t = 15.399	t = 14.651	t = 9.294
			df = 408.155	df = 483	df = 445.501	df = 448.265
			*P* = 0.001	*P* = 0.001	*P* = 0.001	*P* = 0.001

N: number of measured structures.

## Discussion

Cadmium is an environmental pollutant that causes serious toxicity in humans and animals [4]. As a response to this metal toxicity, differences in the weight of various organs were identified after a long-term accumulation of Cd [27,28]. The increase in the femoral weight due to the subchronic exposure to Cd indicates a stimulating effect of this metal on the bone weight. Although Cd is not essential element for growth and development in mammals, it generally follows the metabolic pathways of some essential elements, such as Zn, primarily due to similar physicochemical properties and similar affinities for sulfhydryl groups [29,30]. According to Sigel *et al*. [31], the replacement of Zn^2+^ with Cd^2+^ in many enzymes and transcription factors may induce aberrant gene expression, resulting in the stimulation of cell proliferation. Rat experiments indicate that oral ingestion of Cd through drinking water leads to an accumulation of Cd into the bone as it has also been observed in our study (Cd concentrations in the control and Cd groups were 0.92 ± 0.21 mg/kg dry weight (dw), 1.33 ± 0.18 mg/kg dw, respectively). In accordance with our results, Cd-induced increase in a weight of liver, kidney and spleen has also been mentioned in rats subcutaneously exposed to 2.0 mg CdCl_2_/kg (three days/week or daily) for 28 or 21 days, respectively [27,28]. In contrast, the results obtained by Brzóska *et al*. [18] have shown that the exposure to relatively high doses of Cd (2.073–10.445 mg/kg bw) in drinking water for 12 months resulted in a significantly decreased femoral weight of adult male rats. Therefore, it can be concluded that the impact of Cd on the weight of various organs (including bone) is dose-dependent.

We have found no significant differences for body weight and femoral length between the both groups of rats. Similarly, no demonstrable changes in the body weight [32,33] and femoral length [19] have also been reported in adult male rats after their exposure to 5 or 50 mg Cd/L in drinking water for 12 months.

In general, the thickness of cortical bone is an important parameter in the evaluation of cortical bone quality and strength. According to Garn *et al*. [34], cortical thickness of femoral shaft is a good measuring parameter for evaluation of bone mass. We have found no significant differences in cortical bone thickness between rats from the Cd group and those of the control one. In the study by Comelekoglu *et al*. [13], cortical thickness in the femoral diaphysis was also unchanged in adult female rats after a common low intraperitoneal administration of Cd for 18 weeks.

Our findings from the qualitative histological analysis of compact bone in rats from the control group correspond with those reported by other researches [35-38]. The basic structural pattern of the bone tissue was non-vascular. In addition, some areas of primary vascular radial and/or irregular Haversian bone tissues were observed. However, there was no evidence of true Haversian intracortical bone remodeling in our rats. It is generally known that aged rats and mice lack true Haversian cortical bone remodeling under physiological conditions but not cancellous bone remodeling activity [14,37,39,40]. Therefore, some secondary osteons can be observed in their long bones (mainly near endosteal border). In our study, the newly formed remodeling units within compact bone originated from the endocortical surface and extended deeply into the underlying compact bone. The same results have also been documented in the study of Reim *et al*. [37] for thirteen-month-old male rats.

On the other hand, prolonged intake of moderate dose of Cd induced changes in the middle part of *substantia compacta* (in medial and lateral views) in rats from the Cd group, where primary vascular radial bone tissue was found. Formation of this type of bone tissue may be explained as an adaptive response of the bone to Cd toxicity in order to protect bone tissue against death of cells and subsequent necrosis. It is known that Cd induces apoptosis in osteoblast-like cells [41-43], osteoblasts [44,45] and human tumor-derived Saos-2 osteoblasts [46]. Disappearance of the Haversian canal system, which was replaced by a large quantity of degenerated and necrotic tissue, have been demonstrated in the study by Li *et al*. [47] for ovariectomized rats after a long-term Cd administration.

Additionally, we identified a few resorption lacunae near endosteal surfaces in Cd-exposed rats which signalize an early stage of osteoporosis or a presence of an inflammatory process. However, the inflammatory process is characterized by newly built bone formation on periosteal surface (periosteal apposition) [48] which has not been observed in our bone samples. Therefore, the presence of resorption lacunae in rats from the Cd group could be associated only with an early stage of osteoporosis. Several experimental studies revealed that Cd stimulates the differentiation and activity of osteoclasts and inhibits differentiation and activity of osteoblasts [41,49-51]. These effects cause an uncoupling of the normal balance between bone formation and bone resorption resulting in decreased bone mineral density and increased fracture incidence [52], i.e. typical manifest symptoms of osteoporosis. In addition, it has been demonstrated that Cd can stimulate osteoblasts to secrete prostaglandin E2 *in vitro* [53]. It also increases serum parathyroid hormone levels [54] which are connected with osteoclast formation. Moreover, exposure to low levels of Cd has also been associated with increased urinary Ca excretion and subsequent skeletal demineralization, i.e. effects that may accelerate bone loss and increase bone fragility [55,56].

The vascular (also Haversian) canal constriction identified in rats from the Cd group could be associated with structural changes of blood vessels due to toxic effect of Cd. Each Haversian canal contains blood vessels and nerves supported by loose connective tissue [57]. Blood vessels are also present in vascular canals of primary osteons [58]. In general, the vascular system is a critical target of metal toxicity and actions of metals on the vascular system may play important roles in mediating the pathophysiological effects of metals in specific target organs [59]. It is well known that Cd causes morphological alterations and dysfunction in blood vessels leading to hypertension [60]. According to Martynowicz *et al*. [61], mechanisms of the vascular effect of Cd vary and involve nervous, hormone and intracellular signaling pathways. The results of recent studies [61-63] showed an inhibitory impact of Cd on synthesis and/or releasing of endothelium-derived vasoactive substances (e.g. nitric oxide – NO). Decreased NO concentration in vessels of hypertensive rats can also be associated with increased superoxide anions as a result of Cd-induced oxidative stress [64]. On the other hand, exposure of endothelial cells to Cd significantly increased the secretion of vasoconstrictors, such as angiotensin II and endothelin-I [65]. The important role in physiological as well as pathological processes of bone tissue plays an angiogenesis (i.e. formation of new blood vessels) which is modulated by growth factors, cytokines, adhesion molecules, integrins and enzymes [66]. According to Prozialeck *et al*. [67] and Straub *et al*. [68], angiogenesis has a key role in homeostatic response to various toxicant exposures. Kim *et al*. [60] found that high concentrations of Cd (>10 μm) inhibit vascular endothelial growth factor (VEGF) expression and disrupt the growth of blood vessels. Besides VEGF, adverse effect of Cd on the function of VE-cadherin has also been reported [69]. The results by Kolluru *et al*. [70] demonstrate that Cd can directly inhibit endothelial migration and tube formation. We suppose that all these aspects could contribute to vascular (Haversian) canal constriction in our rats exposed to Cd.

All variables of the secondary osteons had also lower values in Cd-intoxicated rats. Generally, it is known that heavy metals (including Cd) are adsorbed and stored within bones [71]. In bones, Cd may be incorporated into hydroxyapatite (HA) crystals instead of Ca [32,72,73], making them considerably resistant to subsequent dissolution [74]. The results by Blumenthal *et al*. [72] showed that Cd incorporation into the HA introduced little strain in the lattice but resulted in a decreasing C-axis spacing and corresponding crystal size decrease in the C-axis direction. HA crystals, as most important mineral components of bones, are aligned with their long axis parallel to the collagen fiber axis creating concentric lamellae of the secondary osteons [75,76]. On the basis of these findings we speculate that a decrease in HA crystals could partially contribute to the decreased size of the secondary osteons in rats from the Cd group. According to Brzóska and Moniuszko-Jakoniuk [32], the metal incorporated into HA crystals affects consequently bone properties. In respect to altered bone mineral crystal size, bones consisting only small crystals have weakened the mechanical properties of the bone composite [77]. Furthermore, Cd in HA crystals can promote bone resorption and cause osteomalacia or osteoporosis [78,79].

Our study demonstrates that subchronic exposure to Cd had a significant impact on femoral weight and compact bone microstructure in adult male rats. The histomorphometrical changes were identified on levels of primary osteons’ vascular canals, Haversian canals and secondary osteons. Due to increasing trend of Cd environmental contamination and human exposition, our findings are of high importance and could also have practical implications in humans. Our results with the moderate dose of Cd in rats, reflecting possible Cd exposure in humans in specific conditions, give a real indication that the same changes in bone microstructure may also occur in exposed humans. The environmental Cd contamination, as well as improper living habits, such as cigarette smoking, can lead to damages and diseases of bones. Identifying environmental factors (such as Cd) and possible mechanisms that contribute to bone loss, decreased bone vascularization and weakening of mechanical bone properties is the first step to prevention of skeletal damages in humans.

## Conclusions

The current study revealed that subchronic peroral administration of 30 mg CdCl_2_/L in drinking water for 90 days has the significant impact on femoral weight and both the qualitative and quantitative histological characteristics of compact bone in adult male rats. It induces an early stage of osteoporosis, alterations in bone vascularization and potentially diminished bone mechanical properties. Our study can be involved in creation a comprehensive novel insight into bone toxicology in experimental animals.
